# Decreased Oxidative Stress in Male Patients with Active Phase Ankylosing Spondylitis Who Underwent Whole-Body Cryotherapy in Closed Cryochamber

**DOI:** 10.1155/2018/7365490

**Published:** 2018-05-02

**Authors:** Agata Stanek, Armand Cholewka, Tomasz Wielkoszyński, Ewa Romuk, Aleksander Sieroń

**Affiliations:** ^1^Department of Internal Medicine, Angiology and Physical Medicine, School of Medicine with the Division of Dentistry in Zabrze, Medical University of Silesia, Batorego Street 15, 41-902 Bytom, Poland; ^2^Department of Medical Physics, Chelkowski Institute of Physics, University of Silesia, 4 Uniwersytecka St., 40-007 Katowice, Poland; ^3^Department of Biochemistry, School of Medicine with the Division of Dentistry in Zabrze, Medical University of Silesia, Jordana 19 St., 41-808 Zabrze, Poland

## Abstract

**Objective:**

The aim of the study was to estimate the impact of whole body cryotherapy (WBC) on oxidative stress when performed in a closed cryochamber on patients with ankylosing spondylitis (AS).

**Material and methods:**

The effect of ten WBC procedures lasting 3 minutes a day with a subsequent 60-minute session kinesiotherapy on oxidative stress in male AS patients (WBC group *n* = 16) was investigated. To assess the disease activity, the Bath Ankylosing Spondylitis Diseases Activity Index (BASDAI) and Bath Ankylosing Spondylitis Functional Index (BASFI) were calculated. The WBC group was compared to the kinesiotherapy only (KT; *n* = 16) group. The routine parameters of oxidative stress (antioxidant enzymatic and nonenzymatic antioxidant status, lipid peroxidation products, total oxidative status (TOS), and oxidative stress index (OSI)) were estimated one day before the beginning and one day after the completion of the research program.

**Results:**

After the completion of the treatment in the WBC group, a significant decrease of oxidative stress markers (TOS and OSI) and a significant increase of total antioxidant status were observed. The erythrocyte activity of glutathione peroxidase, glutathione reductase decreased significantly in both groups, but the differences of activity of that enzymes prior to post treatment values (Δ) in the KT group were significantly higher as compared to the WBC group. The activity of erythrocyte catalase and plasma ZnCu isoenzyme of superoxide dismutase showed a decreased tendency; erythrocyte total superoxide dismutase activity showed an increased tendency in the WBC group after the completion of the treatment. The BASDAI and BASFI decreased significantly in both groups, but the differences of value indexes prior to post treatment (Δ) were significantly higher in the WBC than KT group.

**Conclusion:**

WBC performed in a closed cryochamber decreases oxidative stress and improves BASDAI and BASFI indexes in male patients during the active phase of ankylosing spondylitis.

## 1. Introduction

Ankylosing spondylitis (AS) is a chronic, usually progressive inflammatory rheumatic disease with severe complications that include sacroiliitis, spondylodesis, peripheral arthritis, and a range of extra-articular manifestations, ultimately leading to impacts upon mobility and societal functioning [1]. The pathogenesis of AS is still unknown, but lately it has been postulated that oxidative stress might be involved in the disease [2–6]. Oxidative stress can induce acute or chronic inflammation through the activation of multiple pathways. When oxidative stress appears as a primary disorder, inflammation develops as a secondary disorder and further enhances oxidative stress [7].

In addition to pharmacological treatment, physiotherapy plays an important role in the treatment of AS patients. They are commonly used to maintain spinal mobility, decrease spinal deformity, and reduce pain as well as improve patient's functioning and quality of life [8, 9].

More and more frequently, whole-body cryotherapy (WBC), as a method of physical medicine, is used in the treatment of rheumatic and inflammatory diseases and muscle spasticity [10]. During WBC treatment, the subject is exposed to extreme cold temperatures (below −100°C) for a short period of time (maximum up to 3 minutes) [11, 12]. The action of cryogenic temperatures causes several favorable physiological reactions such as an analgesic, anti-inflammatory, and a circulatory effect [12–15]. Cryogenic temperatures applied to the whole body, apart from the aforementioned effects, also have significant influence on the psyche, the immune, and endocrine systems [15–18].

However, little is still known about the mechanisms of WBC treatment in AS patients. So far, it has been showed that WBC procedures with subsequent kinesiotherapy may help to decrease pain and inflammatory parameters, improve BASDAI (Bath Ankylosing Spondylitis Diseases Activity Index) and BASFI (Bath Ankylosing Spondylitis Functional Index) indexes, and some spinal mobility parameters [19–21]. It has been also proven that in AS patients, WBC treatment performed in a cryochamber with cold retention may decrease oxidative stress and lipid profile parameters [22].

However, the effect of WBC treatment may depend on the type of cryochamber in which procedures are performed [21, 22]. The most popular cryochambers are chambers with cold retention and closed ones (called Wroclawski type or two-step cryochamber).

In the cryochamber with cold retention, in order to make use of the “cold deposit” phenomenon, the cryogenic chamber is placed about 2.5 m under floor level. It is cooled by synthetic liquid air. The subjects enter the therapy chamber by stairs which constitute a mild adaptive area. An open antechamber (vestibule) is located at the base of the stairs. The vestibule and the proper chamber are separated by double swing doors [23, 24].

The closed Wroclawski-type cryochamber is placed at floor level. It is usually cooled by liquid nitrogen. The vestibule and the proper chamber are located on the same level and separated by a lockable door [24].

But until now, there are no papers in available literature on the impact of WBC treatment performed in a closed cryochamber (Wroclawski type) on oxidative stress in AS patients.

In light of the above findings, and taking account that WBC treatment may be performed in different types of cryochambers, the primary aim of the study was to assess the influence of WBC performed in a closed cryochamber (Wroclawski type) on oxidative stress in AS patients during the active phase.

## 2. Materials and Methods

### 2.1. Subjects

The research was conducted with the consent of the Bioethical Committee of the Medical University of Silesia in Katowice (permission number NN-6501-93/I/07), Poland. All examined subjects provided written informed consent. All investigations were conducted according to the principles expressed in the Declaration of Helsinki (1964).

The research involved a group of 32 nonsmoking male patients with ankylosing spondylitis who were divided randomly by a physician into two groups with an allocation ratio 1 : 1. The first group consisted of 16 AS patients exposed to whole-body cryotherapy procedures with subsequent kinesiotherapy (WBC group, mean age 46.63 ± 1.5 years). The second group consisted of 16 AS patients exposed only to kinesiotherapy procedures (KT group, mean age 45.94 ± 1.24 years). There was no significant difference in the mean age, BMI, BASDAI, BASFI, and treatment between these groups.

Male patients who were successfully enrolled in the research had a definite diagnosis of AS, did not suffer from any other diseases, had no associated pathologies, and had no treatment with disease-modifying antirheumatic drugs (DMARDs), biologic agents, or steroids. The AS patients were treated with doses of nonsteroidal anti-inflammatory drugs (NSAIDs), which were not altered within one month before the beginning of the study and during it. All the patients included in the trial fulfilled the modified New York Criteria for definite diagnosis of AS, which serves as the basis for the ASAS/EULAR recommendations [25]. The final selection for the study included only HLA B27 positive patients, who exhibited II and III radiographic grades of sacroiliac joint disease and attended a consulting unit in a health resort in the period of subsidence of acute clinical symptoms, in order to qualify for physiotherapy treatment. The demographic characteristics of the subjects is shown in Table 1.

Before the research, each patient was examined by a physician to exclude any coexisting diseases as well as any contraindications for WBC procedures. Prior to the research, a resting electrocardiogram was performed in all the patients, and before each session of WBC, the blood pressure was measured for each patient [24].

The patients from both groups were asked to abstain from alcohol, drugs, and any immunomodulators, immunostimulators, hormones, vitamins, minerals, or other substances with antioxidant properties for 4 weeks before the study. All the patients were also asked to refrain from the consumption of caffeine 12 hours prior to laboratory analyses. The diet of the patients was not modified.

### 2.2. Scheme of Whole-Body Cryotherapy and Kinesiotherapy Procedures

Depending on the group, the AS patients were exposed either to a cycle of WBC procedures lasting 3 minutes a day with a subsequent 60-minute session of kinesiotherapy or 60-minute session of kinesiotherapy only for 10 consecutive days excluding the weekend at the same time in the morning.

The WBC procedures were performed in a cryochamber Wroclawski type (closed cryochamber) cooled by liquid nitrogen (produced by Creator, Poland), which consisted of separated two compartments: the antechamber (vestibule) and the proper chamber. The temperature in the antechamber was −60°C, whereas in the proper chamber, it reached −120°C. The subjects entered the chamber in groups of four. Each entry to the cryochamber was preceded by a 30-second adaptation period in the vestibule at −60°C. After adaptation, the subjects stepped into the proper chamber, where they were exposed to cryogenic temperatures for 3 minutes. During the WBC procedure, all the patients were dressed in swimsuits, they wore cotton socks and gloves and wooden shoes, and their mouths and noses were protected by dust masks and their ears by ear-protectors. No jewelry, glasses, and contact lenses were allowed. Each subject was informed about the rules: the need for slow, shallow breathing (short nasal inhalation and longer oral exhalation), and the way to move about (slow walking in circles, one after the other). During the WBC procedure, they were also not allowed to touch each other.

Immediately after leaving the cryogenic chamber, the AS patients underwent kinesiotherapy lasting for one hour. The program of kinesiotherapy was the same for all the patients in both groups. Kinesiotherapy procedures included range-of-motion exercises of the spine and major joints (including the hip, knee, ankle, shoulder, elbow, and wrist). Chest expansion and breathing exercises were also included. Apart from range-of-motion exercise, the AS patients received strengthening exercises of the muscles of the major body parts (spine, arms, and thighs) as well as aerobic exercise (including cycling and fast walking). All the exercises were carried out under the supervision of physiotherapists [22].

All the patients completed the research program and no complications or side effects related to the WBC procedures were observed.

### 2.3. Blood Sample Collection

Blood samples of all the subjects were collected in the morning before the first meal one day before the beginning and one day after the completion of the research program. Samples of whole blood (5 ml) were drawn from the basilic vein and then collected into tubes containing ethylenediaminetetraacetic acid (Sarstedt, S-Monovette with 1.6 mg/ml EDTA-K_3_) and into tubes with a clot activator (Sarstedt, S-Monovette). The blood samples were centrifuged (10 min., 900*g* 4°C) and then the plasma and serum were immediately separated and stored at the temperature of −75°C, until biochemical analyses could be performed. The red blood cells retained from the removal of EDTA plasma were rinsed with isotonic salt solution, and then 10% of the hemolysates were prepared for further analyses. The hemoglobin concentration in the hemolysates was determined by the standard cyanmethemoglobin method. The inter- and intra-assay coefficients of variations (CV) were 1.1% and 2.4%, respectively.

### 2.4. Biochemical Analysis

#### 2.4.1. Oxidative Stress Analysis

*(1) Determination of Activity of Antioxidant Enzymes*. The plasma and erythrocytes superoxide dismutase (SOD-E.C.1.15.1.1) activity was assayed by the Oyanagui method [26]. Enzymatic activity was expressed in nitrite unit (NU) in each mg of hemoglobin (Hb) or ml of blood plasma. One nitrite unit (1 NU) means a 50% inhibition of nitrite ion production by SOD in this method. SOD isoenzymes (SOD-Mn and SOD-ZnCu) were measured using potassium cyanide as the inhibitor of the SOD-ZnCu isoenzyme. The inter- and intra-assay coefficients of variations (CV) were 2.8% and 5.4%, respectively.

The catalase (CAT-E.C.1.11.1.6.) activity in erythrocytes was measured by the Aebi [27] kinetic method and expressed in [IU/mgHb]. The inter- and intra-assay coefficients of variations (CV) were 2.6% and 6.1%, respectively.

The activity of erythrocyte glutathione peroxidase (GPx-E.C.1.11.1.9.) was assayed by Paglia and Valentine's kinetic method [28], with t-butyl peroxide as a substrate and expressed as micromoles of NADPH oxidized per minute and normalized to one gram of hemoglobin [IU/gHb]. The inter- and intra-assay coefficients of variations (CV) were 3.4% and 7.5%, respectively.

The glutathione reductase in erythrocytes (GR-E.C.1.6.4.2) activity was assayed by Richterich's kinetic method [29], expressed as micromoles of NADPH utilized per minute and normalized to one gram of hemoglobin [IU/g Hb]. The inter- and intra-assay coefficients of variations (CV) were 2.1% and 5.8%, respectively.

*(2) Determination of Nonenzymatic Antioxidant Status*. The total antioxidant capacity of plasma was measured as the ferric reducing ability of plasma (FRAP) according to Benzie and Strain [30] and calibrated using Trolox and expressed in [*μ*mol/l]. The inter- and intra-assay coefficients of variations (CV) were 1.1% and 3.8%, respectively.

The serum concentration of protein sulfhydryl (PSH) was measured by Koster et al.'s method [31] using dithionitrobenzoic acid (DTNB) and expressed in [*μ*mol/l]. The inter- and intra-assay coefficients of variations (CV) were 2.6% and 5.4%, respectively.

The serum concentration of uric acid (UA) was assayed by a uricase-peroxidase method [32] on the Cobas Integra 400 plus analyzer and expressed as [mg/dl]. The inter- and intra-assay coefficients of variations (CV) were 1.4% and 4.4%, respectively.

*(3) Determination of Lipid Peroxidation Products, Total Oxidatative Status, and Oxidative Stress Index*. The intensity of lipid peroxidation in the plasma and the erythrocytes was measured spectrofluorimetrically as thiobarbituric acid-reactive substances (TBARS) according to Ohkawa et al. [33]. The TBARS concentrations were expressed as malondialdehyde (MDA) equivalents in [*μ*mol/l] in plasma or [nmol/gHb] in erythrocytes. The inter- and intra-assay coefficients of variations (CV) were 2.1% and 8.3%, respectively.

The serum total oxidant status (TOS) was determined with the method described by Erel [34] and expressed in [*μ*mol/l]. The inter- and intra-assay coefficients of variations (CV) were 2.2% and 6.4%, respectively.

The oxidative stress index (OSI), an indicator of the degree of oxidative stress, was expressed as the ratio of total oxidant status (TOS) to total antioxidant capacity (FRAP) in arbitrary units [35].

### 2.5. Assessment of Activity of Ankylosing Spondylitis

The activity of ankylosing spondylitis was measured by the Bath Ankylosing Spondylitis Diseases Activity Index (BASDAI) and the Bath Ankylosing Spondylitis Functional Index (BASFI).

The BASDAI has six questions related to fatigue, back pain, peripheral pain, peripheral swelling, local tenderness, and morning stiffness (degree and length). Other than the issues relating to morning stiffness, all questions were scored from 0 (none) to 10 (very severe) using a visual analogue scale (VAS). The sum was calculated as the mean of two morning stiffness issues and the four remaining issues [36].

The BASFI is the mean score of ten questions addressing functional limitations and the level of physical activity at home and work, assessed on VAS scales (0 = easy, 10 = impossible) [37].

### 2.6. Statistical Analysis

For statistical analysis, the statistical package of Statistica 10 Pl software was used. For each parameter, the indicators of the descriptive statistics were determined (mean value and standard deviation SD). The normality of the data distribution was checked using the Shapiro–Wilk test, while the homogeneity of the variance was checked by applying the Levene's test. In order to compare the differences between the groups, an independent sample Student *t*-test was used or alternatively the Mann–Whitney *U* test. In the case of dependent samples, the Student *t*-test was used or alternatively the Wilcoxon test. Differences at the significance level of *p* < 0.05 were considered as statistically significant.

## 3. Results

### 3.1. Antioxidants Enzymes

AS patients in the WBC group had, after the completion of treatment, a statistically significant decrease in the erythrocyte activity of GPx (20.6 ± 5.07 and 18.3 ± 4.15 [IU/gHb]—before and after therapy, respectively, *p* = 0.002) and GR (1.21 ± 0.29 and 0.93 ± 0.37 [IU/gHb] [IU/gHb]—before and after therapy, respectively, *p* = 0.007). The erythrocyte activity of CAT (423.0 ± 61.6 and 380.0 ± 102.0 [IU/mgHb]—before and after therapy, respectively, *p* = 0.079) and plasma SOD-CuZn (7.05 ± 1.92 and 5.76 ± 2.36 [NU/ml]—before and after therapy, respectively, *p* = 0.063) showed a decreased tendency. The erythrocyte activity of total SOD (104.0 ± 15.0 and 112.0 ± 11.2 [NU/mgHb]—before and after therapy, respectively, *p* = 0.056) showed an increased tendency.

However, the activity of plasma total SOD (12.4 ± 1.89 and 11.5 ± 3.19 [NU/ml]—before and after therapy, respectively, *p* = 0.501) and SOD-Mn (5.31 ± 1.03 and 6.32 ± 2.16 [NU/ml]—before and after therapy, respectively, *p* = 0.098) did not change significantly in the WBC group after treatment. Similarly, as in the WBC group, the activity of plasma total SOD (12.3 ± 1.85 and 11.7 ± 2.49 [NU/ml]—before and after therapy, respectively, *p* = 0.301) and SOD-Mn (4.56 ± 1.86 and 5.02 ± 1.64 [NU/ml]—before and after therapy, respectively, *p* = 0.642) did not change significantly in the KT group after treatment. In the KT group, the erythrocyte activity of total SOD (128.0 ± 11.2 and 111.0 ± 15.6 [NU/mgHb]—before and after therapy, respectively, *p* = 0.001), GPx (29.9 ± 2.84 and 20.4 ± 5.05 [IU/gHb]—before and after therapy, respectively, *p* = 0.001) and GR (2.07 ± 0.52 and 1.65 ± 0.59 [IU/gHb]—before and after therapy, respectively, *p* = 0.002), similar to the WBC group of patients, decreased significantly after treatment. Additionally, the differences of activity of erythrocyte total SOD (−17.1 ± 11.8 [NU/mgHb] in the KT group versus 7.77 ± 17.2 [NU/mgHb] in the WBC group, *p* < 0.001) and GPx (−9.49 ± 6.74 [IU/gHb] in the KT group versus −2.27 ± 1.98 [IU/gHb] in the WBC group, *p* = 0.001) prior to posttreatment values (Δ) in the KT group were significantly higher as compared to the WBC group. In the KT group, the activity of erythrocyte CAT (425.0 ± 53.6 and 412.0 ± 58.6 [IU/mgHb]—before and after therapy, respectively, *p* = 0.352) and plasma SOD-CuZn (7.80 ± 2.21 and 7.05 ± 3.09 [NU/ml]—before and after therapy, respectively, *p* = 0.326) did not change significantly after treatment in comparison to the WBC group (Table 2).

### 3.2. Nonenzymatic Antioxidant Status

In the WBC group FRAP values (514.1 ± 63.2 and 587.9 ± 50.9 [*μ*mol/l]—before and after therapy, respectively, *p* = 0.001) increased significantly after treatment. The UA level showed an increased tendency in WBC group of patients (4.44 ± 1.43 and 4.75 ± 1.08 [mg/dl]—before and after therapy, respectively, *p* = 0.066). After completion of the treatment FRAP values were significantly higher in the WBC group (587.9 ± 50.9 [*μ*mol/l] when compared to the KT group (499.3 ± 74.6 [*μ*mol/l]) (*p* = 0.001). The level of PSH (627.6 ± 248.0 and 616.5 ± 279.1 [*μ*mol/l]—before and after therapy, respectively, *p* = 0.918) in the WBC group of patients did not change significantly after treatment. In turn, FRP values (550.0 ± 91.3 and 499.3 ± 74.6 [*μ*mol/l]—before and after therapy, respectively, *p* = 0.001) and PSH concentration (393.2 ± 90.0 and 364.7 ± 28.4 [*μ*mol/l]—before and after therapy, respectively, *p* = 0.017) decreased significantly, in the KT group, but the level of UA (4.34 ± 1.15 and 4.61 ± 1.25 [mg/dl]—before and after therapy, respectively, *p* = 0.196) did not change significantly after treatment (Table 3).

### 3.3. Lipid Peroxidation Products, Total Oxidative Status, and Oxidative Stress Index

AS patients in the WBC group had, after the completion of the treatment, a statistically significant decrease in serum TOS (30.49 ± 13.35 and 14.56 ± 9.01[*μ*mol/l]—before and after therapy, respectively, *p* = 0.003) and value of OSI index (64.19 ± 65.93 and 10.20 ± 3.79—before and after therapy, respectively, *p* = 0.001) in comparison to initial values. The differences of these parameters prior to posttreatment values in the WBC group were significantly higher in comparison to the KT group (ΔTOS −15.93 ± 17.04 [*μ*mol/l] in the WBC group versus 0.46 ± 9.11[*μ*mol/l] in the KT group, *p* = 0.003; Δ OSI −53.99 ± 66.83 in WBC group versus 4.78 ± 13.88 in the KT group, *p* = 0.003). The levels of MDA in plasma (2.66 ± 0.73 and 2.48 ± 0.57 [*μ*mol/l]—before and after therapy, respectively, *p* = 0.215) and in erythrocyte (0.16 ± 0.01 and 0.15 ± 0.02 [nmol/gHb]—before and after therapy, respectively, *p* = 0.098) did not change significantly in the WBC group. In the KT group, no significant changes in the levels of plasma MDA (2.32 ± 0.60 and 2.41 ± 0.83 [*μ*mol/l]—before and after therapy, respectively, *p* = 0.959) and erythrocyte MDA (0.18 ± 0.02 and 0.18 ± 0.04 [nmol/gHb]—before and after therapy, *p* = 0.642) as well as serum TOS (23.94 ± 11.60 and 24.41 ± 6.24 [*μ*mol/l]—before and after therapy, respectively, *p* = 0.605) and OSI index (18.87 ± 11.30 and 23.65 ± 15.68—before and after therapy, respectively, *p* = 0.301) were observed after the completion of treatment, in comparison to the initial values before the beginning of the kinesiotherapy cycle (Table 4).

### 3.4. BASDAI and BASFI Indexes

The BASDAI (5.34 ± 1.72 and 3.19 ± 0.91 in the WBC group—before and after therapy, respectively, *p* = 0.001; 5.28 ± 1.71 and 4.53 ± 1.62 in the KT group—before and after therapy, respectively, *p* < 0.001) and BASFI (5.17 ± 2.28 and 3.79 ± 2.21 in the WBC group—before and after therapy, respectively, *p* < 0.001; 5.01 ± 2.06 and 4.35 ± 2.23 in the KT group—before and after therapy, respectively, *p* < 0.001) indexes decreased significantly in both groups, but in the WBC group, with subsequent kinesiotherapy after the completion of the treatment, the decrease of these parameters was significantly higher in comparison to the KT group (Δ BASDAI −2.16 ± 1.29 in the WBC group versus −0.74 ± 0.38 in the KT group, *p* < 0.001; Δ BASFI −1.38 ± 1.07 in the WBC group versus −0.66 ± 0.39 in the KT group, *p* = 0.007). The value of both BASDAI and BASFI indexes was below 4 (inactive phase of AS disease) only in the WBC group after the completion of treatment (Table 5).

## 4. Discussion

After the completion of the treatment, a significant decrease in markers of oxidative stress was achieved in the WBC group of AS patients who underwent a ten-day long cycle of WBC procedures with subsequent kinesiotherapy. In that group of patients, a significant decrease in levels of TOS and OSI values as well as in activity of erythrocyte GPx and GR was observed. A decreased tendency was also noticed in the activity of plasma SOD-CuZn and erythrocyte CAT. Contrary, an increased tendency was seen in the activity of erythrocyte total SOD and the level of UA after the completion of treatment in the WBC group. The FRAP value was increased significantly after the completion of treatment in the WBC group. No significant changes were noted in the activity of plasma total SOD and SOD-Mn, as well as levels of PSH and both plasma and erythrocyte MDA after the completion of treatment in the WBC group.

These findings are similar to our previous research, in which a beneficial impact on oxidative stress from ten WBC procedures performed in a cryochamber with cold retention (temperature: −120°C, time: 3 min, liquid air coolant) both in healthy subjects and AS patients was observed [22, 38]. It has been suggested by Mila-Kierzenkowska et al. [23] that even a single application of cryotherapy prior to exercise may have a beneficial impact on antioxidant system of organism and alleviate the signs of exercise-induced oxidative stress (a single session of WBC, temperature: −130°C, time: 1-2 min., cryochamber with cold retention, liquid air coolant).

However, it has been shown by Lubkowska et al. [39] that a single session of WBC (temperature: −130°C, time: 3 minutes, two-step cryochamber, liquid nitrogen coolant) could induce disturbances in prooxidant-antioxidant balance, in the form of lowering TOS and a temporary decrease in TAS, with a subsequent elevation of those parameters on the following day, resulting in an intensification of oxidative stress.

In another study by the same team [39], healthy men have been exposed to a single WBC session (temperature: −130 ° C, time 3 minutes, two-step cryochamber, liquid nitrogen coolant) without subsequent kinesiotherapy. A significant increase in GPx and GR activities, with a simultaneous decrease in CAT and glutathione S-transferase activities, was observed. A significant increase in the concentration of glutathione, uric acid, albumins, and extra-erythrocyte hemoglobin was also observed in the serum of the subjects. It was concluded by the authors that a single stimulation with cryogenic temperatures results in oxidative stress in a healthy body, but the level of this stress is not very high.

It has been suggested that repeated exposure to cryogenic temperatures may cause adaptative changes in the form of an increase in antioxidant status and antioxidant enzyme activity, resulting in the formation of a prooxidant-antioxidant balance at a higher level (according to hormesis theory) [40].

A beneficial impact of WBC on prooxidant-antioxidant balance has been observed in both male and female kayak athletes when WBC procedures were included into the training regime [40, 41]. The increase in total antioxidant status and the level of UA as a result of a series of short-term whole-body cryotherapy (10 WBC sessions, temperature: −130°C, time: 3 minutes, without subsequent kinesiotherapy) has been also observed by Miller et al. [42] in healthy subjects. However, in other study [43], the authors have shown that the activity of antioxidant enzymes in healthy men depends on the number of WBC procedures (temperature: −130°C, time: 3 minutes). It was also suggested that WBC intensifies oxidative stress and causes an accompanying decrease in antioxidant enzyme activity after 10 sessions, with a subsequent compensatory increase after the completion of a cycle of 20 sessions. In a different study [44], patients with seropositive rheumatoid were observed by the authors to have only a short-term increase in TRAP during the first treatment session of WBC (the temperature− 110°C, three times daily for 7 consecutive days), and no significant oxidative stress or adaptation were caused by the cold treatment.

In our research a significant decrease in BASDAI and BASFI indexes in the WBC group after the completion of treatment was observed. These results are similar to our previous research, in which WBC procedures were performed in a cryochamber with cold retention and two-step cryochamber (Wroclawski type) [19, 22]. In both studies, after the completion of a cycle of WBC procedures consisting of ten 3 minute long WBC procedures daily with subsequent kinesiotherapy (temperature− 120°C, time 3 min, 10 sessions with a weekend break), a decreased of the BASDAI and BASFI index below 4 was observed, which suggests that the AS disease turned into an inactive phase after the completion of treatment. Similar results in AS patients have also been observed by Romanowski et al. [20] (8 daily WBC procedures, the temperature −110°C, time 3 min).

The differences in the results of various studies may be related to the type of cryochamber being used and the coolant medium, in addition to the time of exposure to cryogenic temperatures. Further studies are necessary to estimate whether the number of WBC procedures and type of cryochamber may have an influence on prooxidant-antioxidant balance in subjects who underwent WBC treatment. Comparability of the results obtained by different research teams could be improved through standardization of exposure times and the number of treatments during each cycle. In an attempt to optimise treatment, sexually dimorphism, body fat percentage, and BMI differences should be also taken into account [45, 46].

The present study has some limitations. Firstly, the study did not provide long-term follow-up (at least 3 months), and thus we are not sure how long the beneficial effect of WBC with subsequent kinesiotherapy would be maintained after the completion of a WBC cycle. Secondly, the cycle of WBC with subsequent kinesiotherapy consisted of ten procedures only. A greater number of procedures (e.g., 20–30) could probably intensify the treatment effect. Thirdly, the next studies should involve a larger number of AS patients in different stages of the disease. Females should be also included.

## 5. General Conclusion

Whole-body cryotherapy procedures performed in a closed cryochamber (Wroclawski type) with subsequent kinesiotherapy decrease oxidative stress as well as BASDAI and BASFI indexes in AS patients during the active phase of the disease.

## Figures and Tables

**Table 1:** Demographic characteristics of the research subjects.

Characteristic	WBC group (*n* = 16)	Kinesiotherapy group (*n* = 16)	*p* value
Age, years, mean (SD)	46.63 ± 1.5	45.94 ± 1.24	0.114
Sex M/F	16/0	16/0	—
BMI, kg/m^2^, mean (SD)	24.35 ± 4.4	23.76 ± 6.8	0.968
BASDAI	5.34 ± 1.72	5.28 ± 1.71	0.880
BASFI	5.17 ± 2.28	5.01 ± 2.06	0.940
Smoking (yes/no)	0/16	0/16	—
Medication			
NSAID (yes/no)	16/0	16/0	—
DMARD (yes/no)	0/16	0/16	—
Biological agents (yes/no)	0/16	0/16	—

SD: standard deviation; BMI: body mass index; BASDAI: the Bath Ankylosing Spondylitis Diseases Activity Index; BASFI: the Bath Ankylosing Spondylitis Functional Index; NSAID: nonsteroidal anti-inflammatory drug; DMARD: disease-modifying antirheumatic drug.

**Table 2:** Activities of antioxidant enzymes (mean value ± standard deviation SD) in AS patients before and after the completion of a cycle of ten whole-body cryotherapy procedures with subsequent kinesiotherapy (WBC group) or a cycle of ten kinesiotherapy procedures only (KT group), with statistical analyses. (p): plasma; (e): erythrocyte lysates; Δ: difference prior to post treatment.

Parameters		WBC group	KT group	*p*
Total SOD (p) [NU/ml]	Before	12.4 ± 1.89	12.3 ± 1.85	0.927
	After	11.5 ± 3.19	11.7 ± 2.49	0.884
	*P* ^∗^	0.501	0.301	
	Δ	−0.81 ± 3.08	−0.60 ± 2.65	0.837

SOD-Mn (p) [NU/ml]	Before	5.31 ± 1.03	4.56 ± 1.86	0.170
	After	6.32 ± 2.16	5.02 ± 1.64	0.066
	*P* ^∗^	0.098	0.642	
	Δ	1.01 ± 2.15	0.46 ± 2.46	0.509

SOD-CuZn (p) [NU/ml]	Before	7.05 ± 1.92	7.80 ± 2.21	0.310
	After	5.76 ± 2.36	7.05 ± 3.09	0.194
	*P* ^∗^	0.063	0.326	
	Δ	−1.29 ± 2.46	−0.75 ± 2.72	0.166

Total SOD (e) [NU/mgHb]	Before	104.0 ± 15.0	128.0 ± 11.2	**<0.001**
	After	112.0 ± 11.2	111.0 ± 15.6	0.759
	*P* ^∗^	0.056	**0.001**	
	Δ	7.77 ± 17.2	−17.1 ± 11.8	**<0.001**

CAT (e) [IU/mgHb]	Before	423.0 ± 61.6	425.0 ± 53.6	0.914
	After	380.0 ± 102.0	412.0 ± 58.6	0.294
	*P* ^∗^	0.079	0.352	
	Δ	−42.3 ± 109.0	−13.0 ± 54.0	0.347

GPx (e) [IU/gHb]	Before	20.6 ± 5.07	29.9 ± 2.84	<0.001
	After	18.3 ± 4.15	20.4 ± 5.05	0.207
	*P* ^∗^	**0.002**	**0.001**	
	Δ	−2.27 ± 1.98	−9.49 ± 6.74	**0.001**

GR (e) [IU/gHb]	Before	1.21 ± 0.29	2.07 ± 0.52	**<0.001**
	After	0.93 ± 0.37	1.65 ± 0.59	**<0.001**
	*P* ^∗^	**0.007**	**0.002**	
	Δ	−0.28 ± 0.34	−0.42 ± 0.41	0.275

*P*: statistical significance of differences between both groups of patients; *P*^∗^: statistical significance of differences between values before and after treatment in particular groups of subjects.

**Table 3:** Levels of nonenzymatic antioxidants (mean value ± standard deviation SD) in AS patients before and after the completion of a cycle of ten whole-body cryotherapy procedures with subsequent kinesiotherapy (WBC group) or a cycle of ten kinesiotherapy procedures only (KT group), with statistical analyses. (p): plasma; (s): serum; Δ: difference prior to post treatment.

Parameters		WBC group	KT group	*p*
FRAP [*μ*mol/l	Before	514.1 ± 63.2	550.0 ± 91.3	0.206
	After	587.9 ± 50.9	499.3 ± 74.6	**0.001**
	*P* ^∗^	**0.001**	**0.001**	
	Δ	73.9 ± 55.0	−50.8 ± 39.4	**<0.001**

PSH (s) [*μ*mol/l]	Before	627.6 ± 248.0	393.2 ± 90.0	0.772
	After	616.5 ± 279.1	364.7 ± 28.4	0.239
	*P* ^∗^	0.918	**0.017**	
	Δ	−11.1 ± 281.7	−28.5 ± 92.6	0.605

UA (s) [mg/dl]	Before	4.44 ± 1.43	4.34 ± 1.15	0.838
	After	4.75 ± 1.08	4.61 ± 1.25	0.738
	*P* ^∗^	0.066	0.196	
	Δ	0.31 ± 1.02	0.27 ± 0.70	0.885

*P*: statistical significance of differences between both groups of patients; *P*^∗^: statistical significance of differences between values before and after treatment in particular groups of patients.

**Table 4:** Levels of lipid peroxidation parameters, total oxidative status (TOS), and oxidative stress (OSI) index (mean value ± standard deviation SD) in AS patients before and after the completion of a cycle of ten whole-body cryotherapy procedures with subsequent kinesiotherapy (WBC group) or a cycle of ten kinesiotherapy procedures only (KT group), with statistical analyses. (p): plasma; (s): serum; (e): erythrocyte lysates; Δ: difference prior to post treatment.

Parameters		WBC group	KT group	*p*
MDA (p) [*μ*mol/l]	Before	2.66 ± 0.73	2.32 ± 0.60	0.157
	After	2.48 ± 0.57	2.41 ± 0.83	0.783
	*P* ^∗^	0.215	0.959	
	Δ	−0.19 ± 0.72	0.09 ± 1.04	0.391

MDA (e) [nmol/gHb]	Before	0.16 ± 0.01	0.18 ± 0.02	**0.001**
	After	0.15 ± 0.02	0.18 ± 0.04	**0.001**
	*P* ^∗^	0.098	0.642	
	**Δ**	−0.01 ± 0.03	0.00 ± 0.04	0.155

TOS (s) [*μ*mol/l]	Before	30.49 ± 13.35	23.94 ± 11.60	0.149
	After	14.56 ± 9.01	24.41 ± 6.24	**0.001**
	*P* ^∗^	**0.003**	0.605	
	**Δ**	−15.93 ± 17.04	0.46 ± 9.11	**0.003**

OSI (p/s) [arbitrary unit]	Before	64.19 ± 65.93	18.87 ± 11.30	0.016
	After	10.20 ± 3.79	23.65 ± 15.68	**0.004**
	*P* ^∗^	**0.001**	0.301	
	**Δ**	−53.99 ± 66.83	4.78 ± 13.88	**0.003**

*P*: statistical significance of differences between both groups of patients; *P*^∗^: statistical significance of differences between values before and after treatment in particular groups of patients.

**Table 5:** The value of BASDAI and BASFI indexes (mean value ± standard deviation SD) in AS patients before and after the completion of a cycle of ten whole-body cryotherapy procedures with subsequent kinesiotherapy (WBC group) or a cycle of ten kinesiotherapy procedures only (KT group), with statistical analyses. (p): plasma; (s): serum; Δ: difference prior to post treatment.

Parameters		WBC group	KT group	*p*
BASDAI index	Before	5.34 ± 1.72	5.28 ± 1.71	0.880
	After	3.19 ± 0.91	4.53 ± 1.62	**0.030**
	*P* ^∗^	**0.001**	**<0.001**	
	Δ	−2.16 ± 1.29	−0.74 ± 0.38	**0.001**

BASFI index	Before	5.17 ± 2.28	5.01 ± 2.06	0.940
	After	3.79 ± 2.21	4.35 ± 2.23	0.406
	*P* ^∗^	**<0.001**	**<0.001**	
	Δ	−1.38 ± 1.07	−0.66 ± 0.39	**0.007**

*P*: statistical significance of differences between both groups of patients; *P*^∗^: statistical significance of differences between values before and after treatment in particular groups of patients.

## Data Availability

All data are included in the tables within the article.
